# The importance of underutilized crops for future food and nutrition security

**Published:** 2022-04

**MARCH 24, 2022 -** Staple crops are limited in their tolerance of a changing climate, forcing researchers and breeders to start to investigate new ways to ensure future food security. A review in New Phytologist examines the value of studying underutilized crops, which are locally important crops grown in limited regions, and identifying the specific genes that underpin the crops’ adaptive and valuable traits.

The review demonstrates that extensive genome sequencing is the best way to move from discussions of interesting and unique crops to the breeding of favorable varieties with the potential to move into the mainstream.

The authors note that in the past 20 years, a few previously underutilized crops, such as quinoa, chickpea and pigeonpea, have seen a significant boost in research and recognition. They stress that it is likely that some underutilized crops hold vital genetic variants to help the human population combat food and nutrition insecurity in the next few decades.

“We assembled this review because many underutilized crops have a genome sequenced, but for the most part this has not led to crop varieties in the mainstream,” said author Mark A. Chapman, PhD, of the University of Southampton, in the UK. “The relative ease at which one can sequence plant genomes now means we have the potential to extensively examine the genetics of important traits such as yield and climate tolerance and we advocate this for underutilized crops with potential to combat food insecurity.”

***Link to Study: https://onlinelibrary.wiley.com/doi/10.1111/nph.18021***

***Full citation:** “Beyond a reference genome: pangenomes and population genomics of underutilized and orphan crops for future food and nutrition security” Mark A. Chapman, Yuqi He, Meiliang Zhou. New Phytologist; Published Online: 22 March 2022 (DOI:10.1111/nph.18021).*

*Copyright © 2019 The Cochrane Collaboration. Published by John Wiley & Sons, Ltd., reproduced with permission.*

